# Teaching Transference and Countertransference Across Medicine

**DOI:** 10.1192/j.eurpsy.2026.11966

**Published:** 2026-07-31

**Authors:** E. M. Jetter, D. Valle, H. Nyugen, B. R. Carr

**Affiliations:** 1 College of Medicine; 2 Department of Psychiatry, University of Florida, Gainesville, United States

## Abstract

**Introduction:**

Transference and countertransference, first described by Freud (Freud S. Int J Psychoanal 1912; 12:97-108), reframed by Heimann (Heimann P. Int J Psychoanal 1950; 31:81-84), and elaborated by Racker (Racker H. Transference and Countertransference 1968), are universal in clinical care. Their impact extends beyond psychiatry, as shown by Balint (Balint M. The Doctor, His Patient, and the Illness 1957) and by Melchiode in medical education (Melchiode GA. Am J Psychiatry 1979; 136:1071-1073). Yet while these dynamics shape every encounter, they remain rarely addressed outside psychiatry.

**Objectives:**

To explore how psychoanalytic concepts of transference and countertransference can be applied in medical education to strengthen clinical communication and professional identity formation for physicians in all fields.

**Methods:**

A narrative review of psychoanalytic literature on transference and countertransference was conducted alongside analysis of medical education practices in communication and professionalism. Conceptual integration drew on psychoanalytic teaching traditions to develop strategies for making unconscious dynamics visible and applicable in training.

**Results:**

Introducing transference and countertransference into medical education helps students understand difficult encounters and their own emotional reactions. Teaching strategies to support this integration include (1) Balint-style groups for reflecting on difficult encounters, (2) structured countertransference supervision to help students normalize their reactions, and (3) narrative and reflection exercises linking personal responses to professional identity. These approaches validate emotional experience as a source of clinical insight and resilience.

**Conclusions:**

Teaching transference and countertransference in medical education moves beyond checklist communication skills. It provides learners with a framework for noticing unconscious dynamics, managing their own reactions, and sustaining therapeutic boundaries. Integrating classical psychoanalytic insights with contemporary medical education not only enriches empathy but also supports the resilience and identity formation of future physicians.

**Disclosure of Interest:**

None Declared

